# Whole-genome sequencing rule-out of suspected hospital-onset *Rhizopus* outbreaks

**DOI:** 10.1017/ice.2023.85

**Published:** 2023-12

**Authors:** Victoria T. Chu, Saba Nafees, Eric Waltari, Nicole McNeil, Carolyn Caughell, Estella Sanchez-Guerrero, Lusha Wang, Kim Stanley, Gail Cunningham, Joan Wong, Maíra Phelps, Cristina M. Tato, Steve Miller, Joseph L. DeRisi, Deborah S. Yokoe, Lynn Ramirez-Avila, Charles R. Langelier

**Affiliations:** 1 Division of Infectious Diseases and Global Health, Department of Pediatrics, University of California–San Francisco, San Francisco, California; 2 Chan Zuckerberg Biohub, San Francisco, California; 3 Department of Hospital Epidemiology and Infection Prevention, University of California–San Francisco, San Francisco, California; 4 Division of Infectious Diseases, Department of Medicine, University of California–San Francisco, San Francisco, California; 5 Department of Laboratory Medicine, University of California–San Francisco, San Francisco, California; 6 Illumina, Inc, Foster City, California; 7 Department of Biochemistry and Biophysics, University of California–San Francisco, San Francisco, California

## Abstract

Two independent temporal-spatial clusters of hospital-onset *Rhizopus* infections were evaluated using whole-genome sequencing (WGS). Phylogenetic analysis confirmed that isolates within each cluster were unrelated despite epidemiological suspicion of outbreaks. The ITS1 region alone was insufficient for accurate analysis. WGS has utility for rapid rule-out of suspected nosocomial *Rhizopus* outbreaks.

*Rhizopus* spp are a common etiology of mucormycosis, an invasive fungal infection that affects individuals who are immunocompromised or have diabetes mellitus. Hospital outbreaks secondary to contaminated medical equipment or airborne dissemination of fungal spores have been reported.^1^ From 2020 to 2022, 2 independent temporospatial clusters of hospital-onset *Rhizopus* infections were identified. We used whole-genome sequencing (WGS) to evaluate the genetic similarity of patient isolates and to assess whether the clusters represented hospital outbreaks.

## Methods

Electronic medical records were reviewed for patient history. Hospital-onset was defined as fungal infection diagnosed ≥7 days after admission. Cultures were performed on bronchial alveolar lavage (BAL) or tissue specimens. DNA extraction, library preparation, and Illumina sequencing were performed according to established protocols.^2^ Sequencing reads were assembled and categorized using the Chan Zuckerberg identification (CZID) pipeline.^3^ Alignment of reads to a reference and calculation of distance matrices was performed using the SPID pipeline^4^ with a reference genome from *Rhizopus microsporus* var *rhizopodiformis* strain B11533 (Genbank accession SMRR00000000.1) spanning 27.7 megabases (Mbp) and representing all 27 contigs. Maximum likelihood phylogenetic analysis was performed using Randomized Axelerated Maximum Likelihood (RAxML)^5^ following described methods.^2^ Because ITS1 amplicon sequencing has historically been used to evaluate suspected outbreaks of fungal pathogens,^6^ we assessed whether phylogenetic analysis performed exclusively on this region would yield the same conclusions as WGS. Lastly, we reviewed hospital-onset invasive fungal infection surveillance data from 2018 to 2022 to assess whether there was an increase in fungal infections during this period. The University of California–San Francisco Institutional Review Board granted a waiver of consent for this study, which was part of a larger ongoing surveillance study of patients with outbreak-associated infections (IRB protocol no. 17-24056).

## Results

### Investigation A

Two adult patients (patients A1 and A2) who underwent outpatient bronchoscopy on the same day in October 2020 had rare *Rhizopus* spp in their BAL cultures (Table 1). Patient A1 had diabetes mellitus type 2 and a history of bilateral lung transplantation; patient A2 had a history of lung radiation therapy for gastric cancer and treated pulmonary tuberculosis. BALs were performed for noninfectious evaluations of worsening lung function (patient A1) and mechanical dilation of a chronic airway stricture (patient A2).

**Table 1.** Demographic, Microbiologic, and Sequencing Data for Two Clusters of Patients With Mucormycosis

Patient	Age and Sex	Clinical History	Specimen Source	Culture Results	Species by WGS
**Cluster A: Two adult patients who had an outpatient bronchoscopy procedure performed on the same day at the same hospital with bronchial alveolar lavage (BAL) cultures detecting** ***Rhizopus*** **species.**					
A1	60–70 years, male	History of bilateral lung transplant, type 2 diabetes mellitus, obstructive sleep apnea, obesity, with declining lung function requiring bronchoscopy to evaluate for possible transplant rejection.	BAL	*Rhizopus* spp	*R. microsporus*
A2	50–59 years, female	History of advanced gastric cancer status post gastrectomy, chemotherapy, and lung radiation, and history of treated pulmonary tuberculosis-related chronic airway stricture with recurrent left upper lobe collapse requiring bronchoscopy for mechanical dilation of the airway stricture.	BAL	*Rhizopus* spp	*R. oryzae*
**Cluster B: Three pediatric patients with cutaneous mucormycosis**					
B1	<10 years, male	History of neuroblastoma status post autologous stem cell transplant complicated by refractory transplant associated-thrombotic microangiopathy requiring eculizumab, rituximab, and a 2-week course of methylprednisolone. He subsequently developed a cutaneous mucormycosis cheek lesion under a medical adhesive for a nasogastric tube.	Facial Tissue	*Rhizopus* spp	*R. oryzae*
B2	<10 years, male	History of neuroblastoma status post autologous stem cell transplant complicated by transplant associated-thrombotic microangiopathy requiring eculizimab and high-dose methylprednisolone with development of a large cutaneous mucormycosis cheek lesion under a medical adhesive for an endotracheal tube.	Facial Tissue	*Rhizopus* spp	*R. oryzae*
			Facial Tissue	*Rhizopus* spp	*R. oryzae*
B3	Infant, male	Born at 26 weeks gestational age due to placental insufficiency and anhydramnios. He remained in a humidified incubator due to his extreme prematurity when he developed cutaneous mucormycosis eschar on his abdomen.	Abdominal Eschar	*Rhizopus* spp	*R. microsporus*

Note. WGS, whole-genome sequencing.

**Figure 1.**
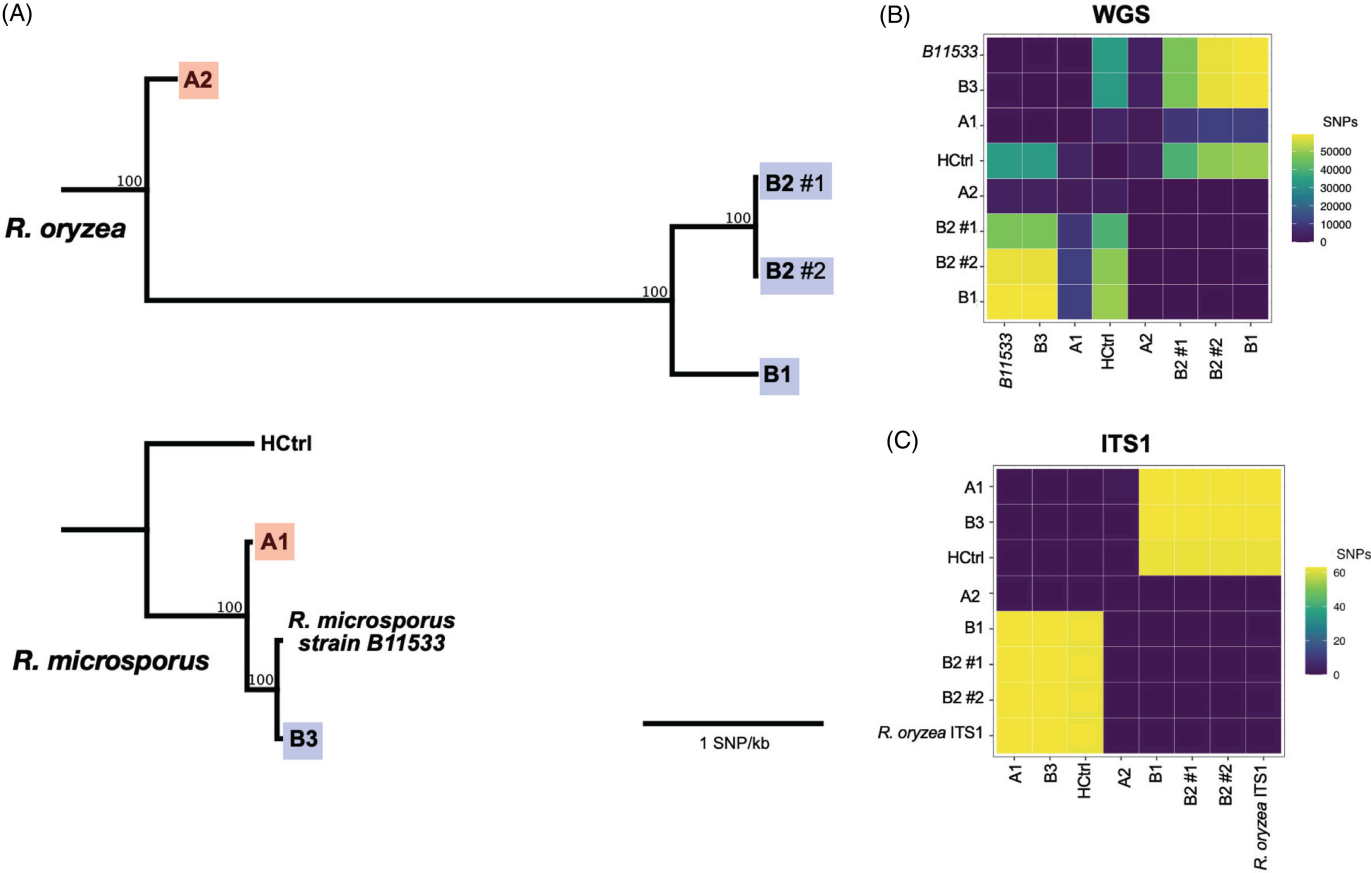
**(**A) Maximum likelihood phylogenetic tree of all 5 patients from *Rhizopus* clusters A and B, based on whole-genome sequencing (WGS), created with RAxML. An historical control (HCtrl) *R. microsporus* isolate was also included. Scale bar represents 1 SNP/kb. (B) Heatmap demonstrating WGS-derived single-nucleotide polymorphism (SNP) distances between isolates. (C) Heatmap demonstrating ITS1-derived SNP distances between isolates, with a *R. oryzae* ITS1 reference.

The unusual occurrence of *Rhizopus* spp in 2 BAL specimens collected on the same day at the same hospital raised concern for a nosocomial infection or contamination in the collection process from a common environmental source that could place other immunocompromised patients undergoing the same procedure at risk for infection. Initial review of the operating room and clinical microbiology laboratory procedures was uninformative other than confirming that the same laboratory staff member plated both BAL samples for culture. WGS and phylogenetic analyses were performed within 48 hours. The isolates were identified as different species: *R. microsporus* (patient A1) and *R. oryzae* (patient A2). They differed by 1,688 single-nucleotide polymorphisms (SNPs) over a shared genome alignment of 0.41 Mbp (Fig. 1). Because WGS identified different *Rhizopus* spp, no further environmental investigation was conducted. Given low clinical suspicion for infection, therapy was not initiated, and no subsequent cultures were obtained.

### Investigation B

Cluster B comprised 3 pediatric patients hospitalized between November 2020 and March 2022 with cutaneous mucormycosis (Table 1). Patients B1 and B2 were autologous stem-cell transplant recipients who developed cheek cutaneous mucormycosis under medical adhesive for a nasogastric tube and an endotracheal tube, respectively. One month after the diagnosis of patient B2 diagnosis, an extremely preterm infant (patient B3) kept in a humidified incubator developed a cutaneous abdominal eschar. Tissue cultures from all 3 patients grew *Rhizopus* spp.

An infection control investigation for each case was launched. It included environmental assessments of the heating and ventilation systems, construction projects, and fungal air sampling. A review of equipment and items with direct contact to the patients’ skin, surface, and bulk cultures of patient-care items was conducted, as well as an analysis of geospatial relationships between the involved patients. The investigations resulted in deep cleaning of patient rooms, changes in equipment maintenance workflow, and a switch in linen vendors after a site visit revealed a lapse in the current vendor’s TRSA certification. However, a common source for the *Rhizopus* was not identified.

WGS was ultimately performed on four isolates among the 3 patients; patient B2 had 2 tissue culture isolates. All 3 patients had distinct *Rhizopus* infections identified as *Rhizopus oryzae* (patient B1, patient B2 isolate 1 and patient B2 isolate 2) and *Rhizopus microsporus* (patient B3) (Fig. 1A). Isolates 1 and 2 from patient B2 each differed from patient B1’s isolate by >600 SNPs over an average shared genome alignment of 0.77 Mbp. Isolates from patients B1 and B2 differed from patient B3’s isolate by >30,000 SNPs over an average shared genome alignment of 3.3 Mbp.

The 2 isolates from patient B2 (obtained independently on the same day) served as a positive control; they were confirmed to be genetically identical (Fig. 1B). An historical isolate from a community-onset *R. microsporus* infection in the same hospital served as a negative control and differed by >2,000 SNPs from the closest investigated isolate (Fig. 1B). The ITS1 region alone distinguished infections from different *Rhizopus* spp. in cluster A, but ITS1 lacked resolution to differentiate between isolates of the same *Rhizopus* spp. in cluster B (Fig. 1C).

Lastly, we asked whether there was an increase in the number of invasive fungal infections at the study site institution between 2018 and 2022. We identified no significant change in the incidence (median, 5 cases per quarter; interquartile range, 4–5 cases per quarter).

## Discussion

An increase in hospital-onset *Rhizopus* cases can reflect sporadic environmental acquisition or active hospital transmission from a point source. Despite epidemiologic, clinical, and microbiologic correlations concerning for nosocomial *Rhizopus* outbreaks or a common environmental source, WGS confirmed that the cases within both clusters were phylogenetically distinct and were likely a result of stochastic occurrences.

Isolates within the suspected clusters differed by >600 SNPs over 3.3 Mbp. Although the number of SNPs needed to distinguish *Rhizopus* strains is undefined, prior studies have suggested that >60 SNPs may reliably distinguish *Rhizopus* strains.^7^ Independent sequencing of 2 positive control isolates from patient B2 showed no SNPs over the shared genome alignment, indicating high analytic reproducibility of the WGS methods used. These investigations support increasing evidence that WGS can be performed rapidly^2^ and can lend clarity to suspected mucormycosis clusters^6^ as well as other invasive fungal outbreaks.^8^

Classically, *Rhizopus* outbreak investigations rely on conventional microbiological methods for species identification.^1^ When sequencing has been employed, it has been primarily restricted to short regions of the genome containing both conserved and variable sequences, such as the ITS1 or 18S regions.^9^ In our study, ITS1 sequencing alone led to a false determination of genetic relatedness between cases, and WGS was needed for the most accurate conclusion.

Using WGS, we were able to conclude that the cases within 2 clusters were unrelated. Prompt resolution of the suspected outbreak in cluster A avoided closure of bronchoscopy suites, sequestration and culturing of bronchoscopes, environmental and air sampling, and time-consuming investigations by hospital infection control personnel. In summary, precision infection control methods incorporating WGS can enable rapid rule-out of suspected *Rhizopus* hospital outbreaks and can complement traditional epidemiologic tools.

## Data Availability

The *Rhizopus* genome sequences associated with this study are publicly available under NCBI BioProject ID PRJNA905128.
